# Individual and setting level predictors of the implementation of a skin cancer prevention program: a multilevel analysis

**DOI:** 10.1186/1748-5908-5-40

**Published:** 2010-05-31

**Authors:** Borsika A Rabin, Eric Nehl, Tom Elliott, Anjali D Deshpande, Ross C Brownson, Karen Glanz

**Affiliations:** 1 Cancer Research Network Cancer Communication Research Center, Institute for Health Research, Kaiser Permanente Colorado, P.O. Box 378066, Denver, CO 80237-8066, USA; 2 Rollins School of Public Health, 1518 Clifton Rd, NE, Emory University, Atlanta, Georgia 30322, USA; 3 Division of Health Behavior Research, Washington University School of Medicine, 4444 Forest Park Ave, Campus Box 8504, St. Louis, MO 63108, USA; 4 Prevention Research Center in St. Louis, George Warren Brown School of Social Work, Washington University in St. Louis, 660 S. Euclid, Campus Box 8109, St. Louis, MO 63110, USA; 5 Department of Surgery and Alvin J. Siteman Cancer Center, Washington University School of Medicine, Washington University in St. Louis, St. Louis, MO 63110, USA; 6 Schools of Medicine and Nursing, University of Pennsylvania, 801 Blockley Hall, 423 Guardian Drive, Philadelphia, PA 19104, USA

## Abstract

**Background:**

To achieve widespread cancer control, a better understanding is needed of the factors that contribute to successful implementation of effective skin cancer prevention interventions. This study assessed the relative contributions of individual- and setting-level characteristics to implementation of a widely disseminated skin cancer prevention program.

**Methods:**

A multilevel analysis was conducted using data from the Pool Cool Diffusion Trial from 2004 and replicated with data from 2005. Implementation of Pool Cool by lifeguards was measured using a composite score (implementation variable, range 0 to 10) that assessed whether the lifeguard performed different components of the intervention. Predictors included lifeguard background characteristics, lifeguard sun protection-related attitudes and behaviors, pool characteristics, and enhanced (*i.e*., more technical assistance, tailored materials, and incentives are provided) versus basic treatment group.

**Results:**

The mean value of the implementation variable was 4 in both years (2004 and 2005; SD = 2 in 2004 and SD = 3 in 2005) indicating a moderate implementation for most lifeguards. Several individual-level (lifeguard characteristics) and setting-level (pool characteristics and treatment group) factors were found to be significantly associated with implementation of Pool Cool by lifeguards. All three lifeguard-level domains (lifeguard background characteristics, lifeguard sun protection-related attitudes and behaviors) and six pool-level predictors (number of weekly pool visitors, intervention intensity, geographic latitude, pool location, sun safety and/or skin cancer prevention programs, and sun safety programs and policies) were included in the final model. The most important predictors of implementation were the number of weekly pool visitors (inverse association) and enhanced treatment group (positive association). That is, pools with fewer weekly visitors and pools in the enhanced treatment group had significantly higher program implementation in both 2004 and 2005.

**Conclusions:**

More intense, theory-driven dissemination strategies led to higher levels of implementation of this effective skin cancer prevention program. Issues to be considered by practitioners seeking to implement evidence-based programs in community settings, include taking into account both individual-level and setting-level factors, using active implementation approaches, and assessing local needs to adapt intervention materials.

## Background

Skin cancer is the most common and one of the most preventable forms of cancer in the United States [1]. An increasing number of effective interventions for the primary prevention of skin cancer are available and recommended; however, few of them have been systematically disseminated and implemented [2]. Furthermore, little is known about the barriers and facilitators to the implementation of effective interventions for the primary prevention of skin cancer [3]. These issues are addressed by the field of implementation research.

Implementation research studies the processes and factors that are associated with and lead to the widespread use and the successful integration of an evidence-based intervention [4]. Implementation of evidence-based interventions most likely occurs in stages and is defined as the process of putting to use an intervention within a specific setting (*e.g*., a school or worksite) [4,5]. The quality of implementation can be characterized by the degree to which the intervention is carried out in a new setting as prescribed by the original protocol (*i.e*., fidelity) [6,7]. Implementation fidelity has been shown to determine the success of the implemented intervention by influencing the relationship between the intervention and the intended outcomes [8,9].

A number of factors influence the speed and extent of implementation of evidence-based interventions, including individual-level and setting-level adopter characteristics, contextual factors, intensity of the intervention, and characteristics of the intervention [9,10]. Characteristics of individuals that influence the implementation include background characteristics (*e.g*., education), attitude toward the intervention, self-efficacy and motivation to implement the intervention, and position within the setting/organization [9]. Attributes of the adopting setting that appear to influence implementation include the setting size, perceived complexity, formalization, and organizational and service system factors (*e.g*., characteristics and style of the leadership, attitude toward the intervention, and administrative and financial support and resources available for the implementation of the intervention) [9,11].

Contextual variables refer to the broader physical, political, social, economic, and historical factors relevant to the implementation [12]. The intensity of the intervention can be characterized by the requisite level of training and technical assistance and the quality of information and materials (*i.e*., tailoring) received by the adopters before and during the implementation [9]. Finally, the perceived characteristics of the intervention affect implementation: these may include relative advantage, compatibility, observability, trialbility, and complexity [4].

Although the role of these factors is well described in the literature [10,13], little research has been done on identifying their relative contributions to the implementation of effective skin cancer prevention interventions. A recent systematic review of the implementation literature found only three skin cancer prevention dissemination and implementation studies published between 1971 and 2008 (excluding the one described and used in this paper) [3,14-16]. The results from these studies regarding factors influencing the implementation process were mixed. Furthermore, these studies did not discuss potential influential factors systematically, did not include a large number of possible predictors, and did not account for the hierarchical structure of these influences (*i.e*., individuals nested within settings). To achieve widespread cancer control, a better understanding is needed of the characteristics that contribute to the successful implementation of effective skin cancer prevention interventions [17].

The analysis reported here addressed an ancillary aim of the Pool Cool Diffusion Trial and assessed the relative contributions of lifeguard background characteristics, sun protective attitudes, sun protective behaviors, pool characteristics, and treatment group to the implementation of a widely disseminated skin cancer prevention program by lifeguards.

### Context

Pool Cool is a multi-component educational and environmental sun safety intervention conducted at swimming pools [18]. Pool Cool was tested in an efficacy trial and found to be effective in improving children's sun protection behaviors, sun safety environments at swimming pool, and reducing sunburns among lifeguards [18,19]. Furthermore, a dose-response relationship was observed between the number of lessons and activities that children were exposed to and their sun protection habits [18].

The efficacy trial was followed by a pilot dissemination study and a larger randomized diffusion trial, the Pool Cool Diffusion Trial. The analysis described in this paper used data from the Pool Cool Diffusion Trial. The Pool Cool Diffusion Trial applied constructs from the social cognitive theory, the diffusion of innovations theory, and theories of organizational change [20], and was designed to evaluate two strategies for the dissemination of Pool Cool. The two dissemination strategies tested in the trial were the basic and enhanced delivery methods (*i.e*., treatment groups). The enhanced group pools received a more intensive, theory-based dissemination intervention, including additional sun safety incentives, more environmental resources, and technical assistance (motivational and reinforcing strategies) in addition to the standard intervention components. More specifically, pools in the basic group received a Pool Cool Toolkit and program training that were similar to the ones used in the original pilot study and efficacy trial [18]. Enhanced pools received the same information and materials as the pools in the basic group plus additional sun-safety resources, including Pool Cool incentive items (hats, UV sensitive stickers, water bottles, *et al*.), additional sun-safety signs, and possibly a shade structure. Pools in the enhanced group were also given booklets entitled, 'How to Make Pool Cool More Effective' and 'The Pool Cool Guide to Sustainability' - a guide that includes suggestions and methods for securing continued funding and support, including developing partnerships with local organizations to continue the program after the end of the research study. Enhanced pools also participated in a 'Frequent Applier' program that earned raffle points as incentives to encourage maximum participation in the program. Raffled items included extra Pool Cool incentive items (hats, lanyards, pens, *et al*.), extra gallons of sunscreen, and shade structures. Field coordinators representing pools from the enhanced group also participated in two to three additional conference calls each summer were actively engaged in discussions regarding program maintenance and sustainability that were not discussed with field coordinators responsible for basic pools.

The Pool Cool Diffusion Trial was conducted across four calendar years for two consecutive cohorts of three years each, starting in 2003 and 2004 at swimming pools in 28 metropolitan areas across the United States. Pools were recruited in cooperation with the National Recreation and Park Association (NRPA) using multiple methods: NRPA web site notices, NRPA email list-serves, conference displays, and targeted advertisements in aquatic magazines and NRPA newsletters. Metro regions were required to have at least a minimum population size of 100,000 and at least four outdoor swimming pools willing to participate. Recruited pools were both public (city, county, military, *et al*.) and private (YMCA, country club, *et al*.). Pools were required to be outdoors, to offer swim lessons to children five to ten years of age, and to be large enough to recruit at least 20 parents to fill out surveys. Lifeguards were not specifically recruited but participated based on their employment at a given study pool. The intervention components, theoretical foundations and examples for each construct, data collection procedures, and findings from the main randomized controlled trial are described in more detail elsewhere [20-23]. The analysis presented in this paper addresses an ancillary aim of the Pool Cool Diffusion Trial that is different from the aims of the main randomized controlled trial.

## Methods

**Figure 1:**
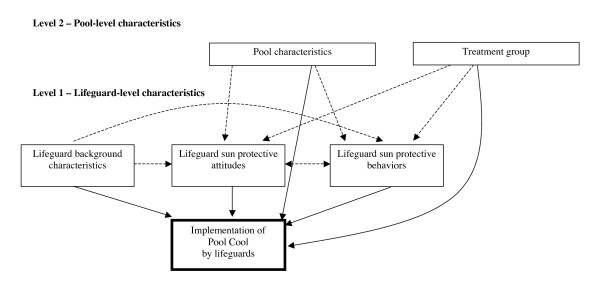
**The effect of individual and setting level characteristics on the implementation of Pool Cool by lifeguards**.

To address the above-described research aim, a multilevel analysis was conducted using a distinct subset of data from the Pool Cool Diffusion Trial from 2004 and 2005. The conceptual framework describing the relationship between different constructs is presented in Figure 1. Lifeguards are believed to play an intermediate role (*i.e*., adopters) in the delivery of the intervention by implementing the educational and certain environmental components of the program. The solid arrows represent relationships that were evaluated in this paper. The dashed arrows indicate existing relationships that were not addressed in this analysis.

### Measures

Data were collected from parents, lifeguards, and pool managers at the beginning (baseline) and at the end (follow-up) of each summer season using self-administered surveys. Data on lifeguard characteristics were obtained from the baseline lifeguard surveys. Items composing the dependent variable ('Implementation of Pool Cool by lifeguards') were from the follow-up lifeguard survey responses, and pool characteristics were identified from baseline pool manager surveys except for one variable (*e.g*., sun safety environments and policies) that was based on the baseline lifeguard survey responses. The variables of interest are shown in Tables 1 and 2.

**Table 1:** Descriptive characteristics for level 2 variables and their origin (n = 288 in 2004 and 287 in 2005)

Variable	2004	2005
	
	% (n)	% (n)
	
Pool characteristics		
North latitude (North or South)	54.90 (158)	48.10 (138)
Urban location (urban or suburban/rural)	37.20 (107)	43.90 (126)
Size of community served		
Less than 50,000	31.60 (91)	26.50 (76)
50,000 to 99,999	24.70(71)	26.50 (76)
100,000 to 299,999	18.80 (54)	16.00 (46)
300,000 or more	25.00 (72)	31.00 (89)
Weekly pool visitors (2,000 or more)	28.10 (81)	27.50 (79)
Pool Manager tenure		
1 year or less	30.90 (89)	35.50 (102)
2 to 4 years	38.50 (111)	34.10 (98)
5 or more years	30.60 (88)	30.30 (87)
	
	**mean (SD)**	**mean (SD)**
	
Sun safety and/or skin cancer prevention programs (1 to 4)*	2.82 (0.83)	2.80 (0.83)
Sun safety environments and policies (1 to 4)*	2.96 (0.74)	3.22 (0.60)
Sun safety and/or skin cancer prevention programs (1 to 4)*	2.82 (0.83)	2.80 (0.83)
**Treatment group**		

Enhanced treatment group (Enhanced or Basic) (%)	51.40 (148)	48.80 (140)

* Possible score range for variable indicated in parenthesis

**Table 2:** Descriptive characteristics for lifeguard variables and their origin (n = 2,704 in 2004 and n = 2,829 in 2005)

Variable	2004	2005
	
	% (n)	% (n)
	
Lifeguard background characteristics		
Female	60.70 (1,640)	59.70 (1,690)
Age (mean (SD))	18.58 (4.63)	18.50 (4.26)
At least college education	36.40 (984)	38.46 (1,088)
Caucasian	89.70 (2,425)	85.40 (2,417)
Skin cancer risk		
Low	26.70 (722)	28.10 (796)
Medium	38.00 (1,028)	37.30 (1,055)
High	35.30 (954)	34.60 (978)
	
	**mean (SD)**	**mean (SD)**
	
**Lifeguard sun protection-related attitudes**		

Sun protective benefits (1 to 4) *	3.53 (0.49)	3.39 (0.49)
Sun protective barriers (1 to 5)*	2.79 (0.63)	2.78 (0.61)
Sun protective norms (1 to 5) *	3.55 (0.83)	3.62 (0.81)
**Lifeguard sun protection-related behaviors**		

Sun protective behaviors (1 to 4)*	2.40 (0.54)	2.49 (0.55)
Sun exposure (1 to 6)*	4.42 (1.33)	4.39 (1.30)
**Dependent variable**		

Implementation of Pool Cool by lifeguards (0 to 10)*	4.00 (2.00)	4.00 (3.00)

* Possible score range for variable indicated in parenthesis and its meaning is discussed in detail in Additional Files 1 and 2

#### Dependent variable

The dependent variable 'Implementation of Pool Cool by lifeguards' measured whether the lifeguard implemented different components of the Pool Cool intervention. The implementation variable had possible scores ranging from 0 to 10 and was created using 16 items from the follow-up lifeguard survey. Items, scoring, and reliability coefficients for the dependent variable are summarized in the Additional File 1.

#### Independent variables

Independent variables of interest included lifeguard background characteristics, lifeguard sun protection-related attitudes, lifeguard sun protection-related behaviors, pool characteristics, and treatment group.

##### Lifeguard variables (level 1)

##### Lifeguard background characteristics

Lifeguard background characteristics included age, gender, education, race, and skin cancer risk. Age was measured as a continuous variable. Education was included as a dichotomous variable (completion of high school versus at least some college). Race was coded as a dichotomous variable (Caucasian or Other). Skin cancer risk measured with four items and risk levels were categorized as low, medium, and high tertiles. Scores and categories were adapted from the Brief skin cancer Risk Assessment Tool (BRAT) developed in a previous study [24]. This score was found to have acceptable to good reproducibility [24].

##### Lifeguard sun protection-related attitudes

Lifeguard sun protection-related attitudes included sun protective benefits, barriers, and norms composite variables [19]. Lifeguard sun protection-related behaviors included sun protective behaviors and sun exposure. These scales were calculated as the mean of non-missing items, when at least half of the scale items were answered. Sun exposure was measured as the daily average number of hours spent in the sun during peak hours (from 10 a.m. to 4 p.m.) [19]. The survey items on sun protection and exposure and sunburn were subject to cognitive testing and results are reported elsewhere [25].

##### Level 2 variables

##### Pool characteristics

Baseline pool manager surveys were used to obtain pool characteristics, except for one variable (*i.e*., sun safety environments and policies). Pool characteristics included latitude, pool location, community size, weekly pool visitors, pool manager tenure, and sun safety and/or skin cancer prevention programs, and sun safety environments and policies variables. The geographical latitude of the pool was coded North if the pool was located north of 37°N and South if the pool was located south of 37°N. Pools were classified according to their location as urban or suburban/rural. The size of the community where the pool is located was measured by the number of residents in the community, as reported by the pool manager, and was classified into four groups: 'Weekly pool visitors' was defined as the number of people admitted to the pool each week during the summer (less than 2,000 visitors versus 2,000 and more visitors), and 'pool manager tenure' was measured by the number of years the pool manager held his position (three groups). The size of the community and pool manager tenure variables were categorized based on their distribution and were included in the multilevel analysis as dummy variables using the lowest category as a reference group. The sun safety and/or skin cancer prevention programs variable was a composite variable based on three questions assessing whether the pool provides different sun safety and/or skin cancer prevention programs and was calculated as the mean of non-missing items when at least two of the three items were answered. The sun safety environments and policies variable was a composite variable calculated as the unweighted sum score for four items and ranged from 1 to 4. The individual items of this composite variable measured whether the pool implemented certain sun safety environmental changes and policies as reported by the lifeguards and originated from the baseline lifeguard survey responses. The composite scores were then aggregate at the pool level using the mean of the score.

All composite scales were computed using items that were designated *a priori *to be scales. To assess internal consistency, Cronbach's α values were computed for the composite variables. The detailed description of the composite variables and the scoring along with the Cronbach's α values are summarized in the Additional File 2.

##### Treatment group variable

The treatment group variable was included as a dichotomous variable determined based on the pool's region which was randomly assigned to enhanced (*i.e*., they received more technical assistance, tailored materials, and incentives) or basic treatment conditions.

##### Data and preliminary analysis

For this analysis, data were obtained from the Pool Cool Diffusion Trial baseline and follow-up lifeguard surveys from 2004 and 2005 and the Pool Cool Diffusion Trial baseline pool manager surveys from 2004 and 2005. Only participants who completed both baseline and follow-up surveys and had complete information for the variables of interest were included in the analysis. Participants with incomplete data sets were excluded from the analyses (n = 329 or 12% in 2004, and n = 220 or 7% in 2005). Attrition analysis was conducted using chi-squared tests and t-tests to compare characteristics of baseline only respondents to those of baseline and follow-up respondents (loss to follow-up: 49.9% in 2004, and 38.8% in 2005) and to compare those with complete and incomplete datasets. Respondents who were excluded from the analysis showed similar characteristics to those who were included (data not shown).

### Statistical analysis

A multilevel analysis was conducted to determine the relative contributions of lifeguard characteristics (level 1) and pool characteristics and treatment group (level 2) to the implementation of Pool Cool by lifeguards. Model building was performed using the data from 2004. To assess the consistency of our findings across data sets, we replicated the final model with the 2005 data. Lifeguard data from 2004 and 2005 were analyzed separately using parallel statistical methods, and the two years' data were treated as replicate studies.

Multilevel analysis was chosen to account for the hierarchical nature of the data (lifeguards nested within pools). Level 1 predictors included lifeguard background characteristics, sun protective attitudes, and sun protective behaviors. Level 2 variables included pool characteristics and treatment group. The multilevel modeling approaches described by Hox [26] and by Raudenbush and Byrk [27] were applied for the analyses. Full maximum likelihood estimation was used for all models. Statistical significance for the model building was determined using an alpha level of 0.05.

#### Null model and model building with level 1 variables

As a first step, a null model was fit to calculate intraclass correlation coefficients (ICCs). The ICC is an indicator of the degree of clustering and is calculated as the proportion of the variance in the dependent variables that is explained by groups (*i.e*., pools) [28]. Second, level 1 predictors were added to the model as fixed effects. Variables from the lifeguard background characteristics, lifeguard sun protection-related attitudes, and lifeguard sun protection-related behaviors domains were entered sequentially as separate blocks. Level 1 continuous variables (*i.e*., age, sun protective barriers, norms, benefits, and behaviors, and sun exposure) were entered centered around the grand mean. The contribution of each block to the model fit was assessed using the change in deviance (-2*log-likelihood) and the Akaike Information Criterion (AIC) parameters. The AIC parameter assesses the goodness-of-fit of a model while it is controlling for its complexity (*i.e*., the number of predictors in the model) [28]. Blocks significantly adding to the model fit (either based on the change in deviance or comparison of AIC values) were retained in the analysis regardless of significance of individual variables within the domain. This approach was taken as variables composing the different domains were included based on theoretical reasoning

#### Model building with level 1 and level 2 variables

Next, level 2 variables were entered stepwise creating random intercepts models. Random intercepts models assume that the level 1 intercept varies across level 2 units (pools), but not the level 1 slopes (effect of level 1 predictor on implementation). The variables were added to the model one at a time (or as a set of dummy variables) and they were retained if they added significantly to the model (*i.e*., chi-square for change in deviance, p-value less than 0.10) or had a statistically significant association with the outcome variable (*i.e*., individual t-ratio, p-value less than 0.05). The level 2 variables were entered into the model in the following order: treatment group, region, community location, community size, weekly pool visitors, pool manager tenure, sun safety and/or skin cancer prevention programs, and sun safety environments and policies.

In the third step, random coefficient models (*i.e*., both level 1 intercept and slope vary randomly across level 2 units) were run for each level 1 variable separately. Significant variance component for the level 1 slope indicated that the effect of the level 1 predictor on the lifeguard participation in Pool Cool (*i.e*., dependent variable) varied across pools. To model this variability, cross-level interactions between the treatment group variable and the level 1 predictor with significant variance component for the level 1 slope were entered to determine whether treatment group assignment accounts for any between-pool variation. Besides coefficient estimates, standardized coefficient estimates were calculated and reported for the final model [26,29].

#### Model for 2005

As indicated earlier, the final model for 2005 was developed by replicating the final model for 2004 with the 2005 data as a parallel model (*i.e*., including the same variables and fixed and random effects). The replication was performed to increase the robustness of the analysis by determining the consistency of the findings across the two data sets.

SPSS 16.0 and HLM 6.0 statistical software programs were used for data management and analysis [30].

## Results

### Descriptive characteristics of the sample

A total of 2,704 lifeguards from 288 pools in 2004 and 2,829 lifeguards from 287 pools for 2005 were included in the analyses. There were an average of 9.39 (SD = 9.18) lifeguards per pool in 2004 and an average of 9.86 (SD = 9.72) lifeguards per pool in 2005. The descriptive characteristics of variables of interest for the pools are summarized in Table 1 and for the lifeguards are summarized in Table 2.

Pools included in the analyses were approximately equally distributed across enhanced and basic treatment groups and north and south latitude and a higher percentage was located in suburban/rural than urban locations and about 28% had less than 2000 visitors weekly in both years.

In both 2004 and 2005, most lifeguards were Caucasian (89.7% in 2004 and 85.4% in 2005), female (60.7% in 2004 and 59.7% in 2005), and had less than college education (63.6% in 2004 and 61.5% in 2005). Lifeguards had a mean age of 18.6 (SD = 4.6) (18.5 (SD = 4.2) in 2005), and spent close to 4.4 hours per day (SD = 1.3 in both years) in the sun during peak hours (between 10 a.m. and 4 p.m.).

Lifeguards scored an average of 4 points (SD = 2 in 2004 and 3 in 2005) on the 'Implementation of Pool Cool by lifeguards' scale. The implementation rate for individual items (items that composed the dependent variable) ranged between 9% and 62%. In 2004, the highest implementation rates were observed for the items indicating whether the lifeguard used the sunscreen from the large dispenser (62%), received sunscreen samples (50%), taught the Pool Cool sun safety lessons at least once (45%), and knew where the Pool Cool's Leader's Guide was kept at the pool (42%) and used it (38%). The lowest implementation rates were found for the items indicating whether the lifeguard received a t-shirt (9%) or participated in the sun protective clothing (15%) and the colored sunscreen demonstration (17%) activities. Similar items had the highest implementation rates in 2005, including items indicating whether the lifeguard used the sunscreen from the large dispenser (63%), taught the Pool Cool sun safety lessons at least once (55%), received sunscreen samples (52%) and message pen (48%), knew where the Pool Cool's Leader's Guide was kept at the pool (41%), and used it (38%). In 2005, the lowest implementation rates were found for the items indicating whether the lifeguard received a t-shirt (12%), and participated in the Sun Jeopardy game (14%) and sun protective clothing activities (16%).

### Multilevel analysis

The final models for 2004 and 2005 are summarized in Tables 3 and 4. The ICC values calculated from the level 1 and level 2 variances of the fully unconstrained null model were 0.35 in 2004 and 0.34 in 2005 indicating that pool-level variables accounted for 35% (34% in 2005) of the variance in program implementation by lifeguards.

**Table 3:** Final model for lifeguard-level and pool-level predictors of Lifeguard Pool Cool participation for 2004 analysis

Variable	Coefficient	Standardized coefficient	p value
**Intercept**	4.134		0.000
**Level 1 predictors**			
**Lifeguard background characteristics**			
Female	0.212	0.043	0.014
Age	0.023	0.044	0.052
At least some college education	0.451	0.090	0.000
**Lifeguard sun protection-related attitudes**			
Sun protective benefits	0.198	0.040	0.023
Sun protective barriers	0.019	0.005	0.777
Sun protective norms	0.064	0.022	0.293
**Lifeguard sun protection-related behaviors**			
Sun protective behaviors	0.212	0.048	0.011
Sun exposure	0.145	0.080	0.000
**Level 2 predictors**			
Pool characteristics			
North region	-0.233	-0.049	0.172
Urban location	0.366	0.073	0.042
Weekly pool visitors (2,000 or more)	-0.969	-0.182	0.000
Sun safety and/or skin cancer prevention program	0.207	0.072	0.056
Sun safety environments and policies	0.309	0.095	0.025
Treatment group			
Enhanced treatment group	0.617	0.129	0.001
			
Model fit	**Deviance**	**Param**	**AIC**
	11,604.87	22	11,648.87

**Table 4:** Final model for lifeguard-level and pool level predictors of Lifeguard Pool Cool participation for 2005 analysis

Variable	Coefficient	Standardized coefficient	p value
**Intercept**	3.924		0.000
**Level 1 predictors**			
**Lifeguard background characteristics**			
Female	0.389	0.069	0.000
Age	0.063	0.056	0.000
At least some college education	0.362	0.064	0.001
**Lifeguard sun protection-related attitudes**			
Sun protective benefits	0.091	0.016	0.285
Sun protective barriers	0.088	0.019	0.228
Sun protective norms	0.014	0.004	0.825
**Lifeguard sun protection-related behaviors**			
Sun protective behaviors	0.407	0.073	0.000
Sun exposure	0.163	0.076	0.000
**Level 2 predictors**			
Pool characteristics			
North region	0.607	0.110	0.002
Urban location	0.053	0.010	0.791
Weekly pool visitors (2,000 or more)	-1.177	-0.191	0.000
Sun safety and/or skin cancer prevention program	0.112	0.033	0.362
Sun safety environments and policies	0.481	0.104	0.006
Treatment group			
Enhanced treatment group	0.730	0.131	0.000
			
Model fit	**Deviance**	**Param**	**AIC**
	12902.36	22	12,946.36

#### Model building with level 1 predictors (2004 data)

The sub-models for the level 1 domains for 2004 are presented in Additional File 3. All three lifeguard-level (level 1) predictor domains (entered in the order of lifeguard background characteristics, lifeguard sun protective attitudes, lifeguard sun protective behaviors) contributed significantly to the model as shown by both the decrease in deviance and AIC values (Models 1 through 3). Initially all predictors (regardless of individual statistical significance) were kept in the model. However, because unlike the other domains, the lifeguard background characteristics domain was constructed with less theoretical rigidity, sensitivity analysis was conducted to determine whether nonsignificant lifeguard background characteristics predictors (*e.g*., race and skin cancer risk) significantly added to the model. The model with all predictors (Model 3) and the model without nonsignificant lifeguard background characteristics predictors (Model 4) were compared using the change in deviance and AIC values. These values both showed that the two variables did not significantly improve the model fit, hence the more parsimonious model (Model 4) was selected for further model building.

#### Model building with level 1 and level 2 predictors (2004 data)

Level 2 predictors were added one by one or as a set of dummy variables and retained in the model if they met the criteria described in the Methods section of this paper. After identifying the final random intercept model with level 1 and level 2 predictors, random coefficient models were created on a variable-by-variable basis. The variance components for sun protective norms and age were statistically significant, suggesting that the association between sun protective norms and the implementation of Pool Cool and age and the implementation of Pool Cool varied across pools. When including both sun protective norms and age as random effects, neither of the variance components remained statistically significant. However, the change in deviance and AIC values comparing the final random intercept model and the model with random coefficient for sun protective norms and age both indicated that the inclusion of the random effects for these two variables improved the model. Therefore, they were kept as random effects in the model.

When treatment group was added as a level 2 predictor for the sun protective norms and age slopes separately, neither of the cross-level interactions was statistically significant, suggesting that treatment group does not explain the variation in slope for sun protective norms or age (*i.e*., treatment group does not explain the variation in the effect of sun protective norms or age on implementation) (data not shown).

#### Final model for 2004

The final model with random slopes for sun protective norms and age variables is summarized in Table 3. The intercept coefficient in the final model was 4.13, indicating that a male lifeguard with high school education or less, and with mean values for age, barriers, benefits, norms, behaviors, sun exposure, and sun safety environments and policies from a pool from a south region, suburban/rural location, who received basic intervention, had less than 2,000 visitors weekly had an average implementation score of about 41%.

All significant lifeguard background characteristics (female gender, age, education) were positively associated with implementation of Pool Cool. All three predictors (sun protective benefits, barriers, and norms) from the lifeguard sun protection-related attitudes domain also were directly associated with the implementation of Pool Cool, but this association was not statistically significant for the sun protective barriers and norms variables. Both sun protective behaviors and sun exposure showed statistically significant positive associations with implementation. From the pool-level predictors, enhanced treatment group, urban location, sun safety and/or skin cancer prevention programs, and sun safety environments and policies were positively associated and north region and weekly pool visitors were inversely associated with the implementation of Pool Cool. In the final model, north region was no longer a statistically significant association with the outcome.

After standardizing the coefficients, the magnitudes of the slopes suggest that the number of weekly pool visitors had the strongest (inverse) association with the implementation of Pool Cool, closely followed by the treatment group variable (positive association).

#### Final model for 2005

To evaluate the consistency of findings across years, the final model from 2004 was fit to the 2005 data. The main results of the replication were comparable to the 2004 results with a few exceptions. For the sun protection-related attitudes domain, the sun protective benefits coefficient was also nonsignificant, and the sun protective norms variable was inversely associated with the implementation of Pool Cool. For the pool characteristics, region had a statistically significant inverse association with the outcome (with north region having lower implementation), and the coefficients for location and sun safety and/or skin cancer prevention programs were nonsignificant. Similar to the 2004 results, the standardized coefficients indicated that the number of weekly pool visitors followed by treatment group had the strongest associations with implementation of Pool Cool (Table 4).

## Discussion

This study used multilevel methods to evaluate the relative contributions of lifeguard-level and setting-level adopter characteristics and treatment group to the implementation of an effective and widely disseminated skin cancer prevention intervention. Several individual-level (lifeguard characteristics) and setting-level (pool characteristics and treatment group) factors were found to be significantly associated with implementation. The most important predictor of implementation was the number of weekly visitors (inverse association) at the pool, closely followed by enhanced treatment group (positive association).

A common measure of the quality and success of implementation is the degree of implementation [8]. In the context of this study, the degree of implementation was measured by a composite score calculated based on the level of implementation of Pool Cool intervention components by lifeguards, on a scale ranging from 0 to 10. The mean value on this scale was four (SD = 2 in 2004 and 3 in 2005) in both years (2004 and 2005) indicating moderate implementation for most lifeguards. The individual items that were implemented most often were the ones that indicated whether the lifeguard used sunscreen, received sunscreen sample or a message pen, taught the Pool Cool sun safety lessons, and knew the location of and used the Pool Cool's Leader's Guide. These are considered main components at the core of the Pool Cool program [23].

The intraclass correlation for pools in these data was relatively high (35% in 2004 and 34% in 2005), which underscores the usefulness of a multilevel approach in analyzing the data. It also indicates that about 35% of variance in implementation is explained by level 2 characteristics.

All three lifeguard-level domains significantly contributed to the variance in implementation. Education was the most important level 1 predictor of implementation, suggesting that lifeguards with at least some college education were more likely to implement Pool Cool than lifeguards with a high school education or less. This finding is consistent with conclusions from previous studies showing higher levels of education to higher implementation levels among the adopters [6,13,31].

The adopters' positive attitude toward and their self-efficacy to implement an intervention have been shown to increase the likelihood of successful implementation of evidence-based interventions [9,32,33]. Furthermore, previous implementation research in the physical activity literature found that if the delivery agents themselves practiced the health behavior promoted by the intervention, they were more likely to successfully implement the program [34-37]. In this study, both lifeguard sun protection-related attitudes and sun protection-related behaviors significantly explained variance in implementation, although the individual predictors of sun protective barriers and norms had nonsignificant coefficient estimates. This instability might explain the unexpected, positive relationship between sun protective barriers and implementation.

Six level 2 predictors were included in the final model (number of weekly pool visitors, intervention intensity, latitude, pool location, sun safety and/or skin cancer prevention programs, and sun safety programs and policies), three of which (weekly pool visitors, sun safety environments and policies, and intervention intensity) showed consistent direction of effect and statistical significance across the two years.

The most important predictor of implementation in the final model was the number of weekly pool visitors. In this study, an inverse relationship was observed between the number of weekly pool visitors and the level of implementation for Pool Cool by lifeguards. This variable is a proxy for the size of the pool and might influence implementation fidelity in a number of ways. The most likely explanation for the inverse correlation between the number of weekly pool visitors and implementation is that because pools received the same amount of intervention materials regardless of their size, implementation might have been more limited in larger pools where lifeguards had to share the same amount of resources for more visitors. This explanation suggests that, to increase implementation of the intervention, the amount of intervention materials provided for the pools should be proportional to the number of visitors the pools serve.

There is a growing agreement among researchers and practitioners that more innovative and active approaches enhance the implementation of effective interventions [36,38-40]. More intensive implementation strategies include but are not limited to tailoring and packaging of the intervention materials in a user-friendly way, enhancing organizational capacity, establishing systems and rewards for implementation, providing training and technical assistance to adopters, and conducting and reporting evaluation of implementation efforts [9,16,33,41-43]. For example, a study by Mueller and colleagues [44] that evaluated the effectiveness of different strategies for the dissemination of evaluation results on tobacco control programs to program stakeholders found that multi-modal and more active approaches to dissemination increased the usefulness and further dissemination of the evaluation results. Furthermore, previous implementation research studies of skin cancer prevention found mixed results on the effect of intensity of intervention [14-16]. For example, Schofield and colleagues were assessing two strategies for the dissemination of a sun-protection policy in primary and secondary schools in New South Wales, and found that more intensive implementation strategies were more effective in primary schools but not in secondary schools [14]. In a study conducted by Buller and colleagues using web-based strategies to disseminate a sun safety curriculum to elementary schools and child care facilities, intensity of the intervention (basic versus enhanced website) did not seem to influence the online purchase of the program [15]. Finally, Lewis and colleagues disseminated a sun safety program to zoological parks and found that more intense implementation strategies resulted in only marginally significant improvement in short-term implementation for certain components of their intervention and no difference was observed for long-term implementation when compared to the basic implementation approach [16].

In our analysis, treatment group was the second most important predictor of implementation levels. Lifeguards at pools that were randomized to the enhanced treatment group implemented the intervention more than did pools that received the basic treatment. Similar results were found for each subscale of the dependent variable in a post hoc analysis. These findings reinforced the role of more active, multi-component strategies in successful implementation.

Although there were more nonsignificant variables at level 2 (pool characteristics) in 2005 than in 2004, the final models across these two years were consistent. Overall, the patterns in the 2005 final model were similar to the findings from the 2004 analysis and the replication analysis confirmed the robustness of weekly pool visitors and intervention intensity as important predictors of implementation of Pool Cool.

To our knowledge, this is the first skin cancer prevention implementation study using clustered randomized controlled design, including a large number of potential influencing factors and accounting for their multilevel nature. Furthermore, the large sample size and use of two years worth of data with replicate analyses make the findings from this study a robust addition to the existing implementation research literature.

Several limitations of this study should be acknowledged. First, close to 50% of baseline respondents in 2004 and 40% of baseline respondents in 2005 were excluded from the final analysis due to inability to identify the matching follow-up survey responses. During data management, efforts were made to include as much data as possible and to compare baseline information for included and excluded surveys. In order to keep the lifeguard surveys brief, lifeguard perceptions of the intervention characteristics were not measured in the Pool Cool Diffusion Trial. However, extensive information was already available on the acceptability of the Pool Cool program and on the program-related factors that contributed to the implementation of the intervention (*e.g*., ease of program implementation, compatibility of program with swim lessons, comments about major program components) from the pilot study, the efficacy trial, and the process evaluation of the Pool Cool Main Trial and the pilot study of the Pool Cool Diffusion Trial (results are reported elsewhere) [18,45]. Finally, Pool Cool is a multi-component intervention, and it is not possible to separate out the effects of influencing factors on different components. However, the health behavior literature suggests that in the context of complex, multi-component interventions, the measurement of implementation fidelity should focus on the functions and process of the intervention rather than on the individual components [46].

## Summary

The most noteworthy finding from this analysis is that enhanced treatment group was associated with greater implementation of skin cancer prevention interventions-- indicating that more intense, theory-based strategies can lead to higher levels of implementation. Future analyses will examine the most important predictors of change in sun protective behaviors and sunburns (*i.e*., outcomes) among the ultimate target audience of Pool Cool (*i.e*., children) and whether higher implementation levels lead to better outcomes.

Findings from this analysis of a skin cancer prevention intervention are applicable to other public health promotion and prevention areas and suggest several issues that should be considered by practitioners seeking to implement evidence-based programs in community settings, including:

1. Both individual-level and setting-level factors should be considered to enhance implementation of evidence-based interventions.

2. Practitioners should use active implementation approaches including multiple channels, ongoing technical assistance, and tailored materials when implementing evidence-based interventions.

3. It is necessary to assess local needs and adapt the intervention materials accordingly (*e.g*., larger settings may require more resources).

To achieve the widespread use of effective evidence-based interventions, we have to better understand which factors contribute to the successful implementation of these programs. This study makes a valuable contribution to the limited knowledge in this area by identifying factors that can enhance the use of effective programs which will ultimately lead to larger public health effect.

## Competing interests

The authors declare that they have no competing interests.

## Authors' contributions

BAR carried out data management, analysis of the data including multilevel modeling, interpretation of data, and created the first draft of the manuscript. EN was involved with the management of data, participated in the analysis and interpretation of data, and provided revisions on the content of the manuscript. TE coordinated the original data collection and was involved with the data management. ADD was involved with the data analysis (with a special focus on multilevel modeling) and participated in the interpretation of data. She also provided revisions on the content of the manuscript. RCB was involved with the initial conception and design of the analysis and was involved with the data analysis and interpretation and provided revisions on the content of the manuscript. KG led the original conception, design, and acquisition of the data for the Pool Cool Diffusion Trial, supervised the data management and analysis, and participated in the interpretation of data. She also provided revisions on the content of the manuscript. All authors read and approved the final manuscript.

## Supplementary Material

Additional file 1 **Items, scoring, and Cronbach's reliability coefficients for dependent variables**. This pdf file includes information about the items composing the dependent variable of Pool Cool implementation by lifeguards, the scoring used to calculate this composite variable, and the Cronbach's reliability coefficients calculated for each subscale and the composite variable. Click here for file

Additional file 2 **Items, scoring, and Cronbach's reliability coefficients for independent scales**. This pdf file includes information about the items composing a number of independent variables, the scoring used to calculate these composite variables, and the Cronbach's reliability coefficients calculated for each sub-scale and the composite variables. Click here for file

Additional file 3 **Multilevel model results with Level 1 predictors for 2004**. This pdf file provides the coefficient estimates and other model-related information for the sub-models (Models 1 through 4) created using level 1 domains. Click here for file
