# Supplementary material for: Individual and setting level predictors of the implementation of a skin cancer prevention program: a multilevel analysis
Source: Implement Sci. 2010 May 31;5:40. doi: 10.1186/1748-5908-5-40 (PMC2901365; doi:10.1186/1748-5908-5-40)
Supplement: Additional file 1 — Items, scoring, and Cronbach's reliability coefficients for dependent variables. This pdf file includes information about the items composing the dependent variable of Pool Cool implementation by lifeguards, the scoring used to calculate this composite variable, and the Cronbach's reliability coefficients calculated for each subscale and the composite variable. [file 1748-5908-5-40-S1.PDF]

Additional file 1: Items, Scoring and Cronbach's Reliability Coefficients (α) for the dependent variable

Index name (Number of items) and Component items	Range of possible scores	Cronbach's α
**<u>Implementation of Pool Cool by lifeguards</u>**<br>(16 items)	0-10<br>Index score = sum of all the recoded items.	0.76
**Pool Cool Activities** (5 items)<br>Did you take part in these Pool Cool Poolside Activities?<br>1. Weather Watch: The UV Index<br>2. Colored Sunscreen Demonstrations (Blue & Purple People)<br>3. Sun Jeopardy game<br>4. Sun protective clothing activity (Emperor's Clothes)<br>5. Sun exposure card and UV Warning Patch Activity	0-2<br>Answer: Yes/ No<br>Index score: sum of yes answers<br><br>Recode of index score:<br>0 = 0<br>1,2 = 1<br>3,4,5 = 2	0.82
**Pool Cool Leader's Guide** (2 items)<br>1. Did you use the Pool Cool Leader's Guide?<br>2. Do you know where the Pool Cool Leader's Guide is kept at this pool?	0-2<br>Answer: Yes/No<br>Index score: sum of yes answers.	0.62
Did you use the sunscreen in the large dispenser at your pool?	0-1<br><br>Answer: Yes/No<br>Index score: sum of yes answers.	N/A

Additional file 1: Continued

Index name (Number of items) and Component items	Range of possible scores	Cronbach's α
How many times in total did you teach the Pool Cool sun safety lessons to children?	0= I do not teach swimming lessons, I didn't teach because of age of the group, or I was not able to teach the class of Pool Cool.<br>1 = 1-4 times<br>2 = 5-8 times,<br>3 = >8 times	N/A
**Pool Cool Items** (7 items)<br>Did you receive any of these Pool Cool Items?<br>1. Sunscreen samples<br>2. Water bottle<br>3. Lanyard<br>4. Message pen<br>5. UV Meter Card<br>6. UV Warning Patch and Wristband<br>7. T-shirt	0-2<br><br>Answer: Yes/No<br>Index score: sum of yes items<br><br>Recode of index score:<br>0 = 0<br>1,2 = 1<br>3,4,5,6,7 = 2	0.83
