# Supplementary material for: Individual and setting level predictors of the implementation of a skin cancer prevention program: a multilevel analysis
Source: Implement Sci. 2010 May 31;5:40. doi: 10.1186/1748-5908-5-40 (PMC2901365; doi:10.1186/1748-5908-5-40)
Supplement: Additional file 2 — Items, scoring, and Cronbach's reliability coefficients for independent scales. This pdf file includes information about the items composing a number of independent variables, the scoring used to calculate these composite variables, and the Cronbach's reliability coefficients calculated for each sub-scale and the composite variables. [file 1748-5908-5-40-S2.PDF]

Additional file 2: Items, Scoring and Cronbach's reliability coefficient (α) for the skin cancer risk, sun protective barriers, benefits, norms, behaviors, sun safety environments and policies, and sun safety and/or skin cancer prevention programs scales

Index name and (No. of items) and Component items	Range of possible scores	Cronbach's α
**Skin cancer risk** (5 items)<br>1. What is your natural hair color?	Answers:<br>1. Red<br>2. Blond<br>3. Light brown<br>4. Dark brown<br>5. Black<br><br>Recoding of answers:<br>1 = 4<br>2 = 3<br>3 = 2<br>4 = 1<br>5 = 0	0.45
2. What is the color of your eyes?	Answers:<br>1. Green<br>2. Blue<br>3. Light brown<br>4. Dark brown<br>5. Black<br><br>Recoding of answers:<br>1 = 4<br>2 = 3<br>3 = 2<br>4 = 1<br>5 = 0	

Additional file 2: Continued

Index name and (No. of items) and Component items	Range of possible scores	Cronbach's α
3. As a child did you have more than one severe sunburns? (i.e., painful and/or blistering)	Answers:<br>1. Yes<br>2. No<br><br>Recoding of answers:<br>1 = 3<br>2 = 0	
4. What is the color of your untanned skin?	Answers:<br>1. Very fair<br>2. Fair<br>3. Olive<br>4. Dark<br>5. Very dark<br>6. Black<br><br>Recoding of answers:<br>1 = 20<br>2 = 18<br>3 = 16<br>4 = 4<br>5 = 2<br>6 = 0	

Additional file 2: Continued

Index name and (No. of items) and Component items	Range of possible scores	Cronbach's α
5. After being in direct sunlight for more than 30 minutes, do you get:	Answers:<br>1. A severe burn with blistering<br>2. A severe burn without blistering<br>3. A mild burn but then tan or darken<br>4. Tan slowly<br>5. Tan easily<br><br>Recoding of answers:<br>1 = 3<br>2 = 3<br>3 = 2<br>4 = 1<br>5 = 0<br><br>Index score: sum of recoded items (0-34)<br><br>Recode of index score:<br>Lowest thru 21 = Low risk<br>22-25 = Medium risk<br>26 thru highest = High risk	

Additional file 2: Continued

Index name and (No. of items) and Component items	Range of possible scores	Cronbach's α
**Sun protective benefits** (7 items)<br><br>It helps to…<br>1. Use sunscreen<br>2. Wear a shirt with sleeves<br>3. Wear a hat<br>4. Wear sunglasses<br>5. Have a good base suntan<br>6. Stay in the shade or under a beach umbrella<br>7. Limit the number of hours outdoors when the sun's rays are the strongest, at mid-day	Mean of nonmissing items when at least half of the items were answered<br><br>Answers:<br>1 – Not at all<br>2 – A little<br>3 – Somewhat<br>4 – A great deal	0.73
**Sun protective barriers** (3 items)<br><br>How much do you agree with the following?<br>1. People are more attractive if they have a tan<br>2. It's too much bother to put on a hat when you go outside<br>3. I find it difficult to protect myself from the sun.	Mean of nonmissing items when at least half of the items were answered<br><br>Answers:<br>1 – Strongly disagree<br>2 – Disagree<br>3 – Neutral<br>4 – Agree<br>5 – Strongly agree	0.41

Additional file 2: Continued

Index name and (No. of items) and Component items	Range of possible scores	Cronbach's α
**<u>Sun protective norms</u>** (3 items)<br><br>How much do you agree with the following statements?<br>1. Most of the lifeguards I know use sunscreen as protection from the sun when they are outdoors.<br>2. Most of the lifeguards I know wear hat as protection from the sun when they are outdoors.<br>3. Most of the lifeguards I know cover up to protect themselves from the sun.	Mean of nonmissing items when at least half of the items were answered<br><br>Answers:<br>1 – Strongly disagree<br>2 – Disagree<br>3 – Neutral<br>4 – Agree<br>5 – Strongly agree	0.71
**<u>Sun protective behaviors</u>** (5 items)<br><br>When you are outdoors in the sun, how often do you do the following?<br>1. Wear a shirt with sleeves<br>2. Wear sunglasses<br>3. Stay in the shade or under an umbrella<br>4. Wear sunscreen<br>5. Wear a hat	Mean of nonmissing items when at least half of the items were answered<br><br>Answers:<br>1 – Rarely or never<br>2 – Sometimes<br>3 – Usually<br>4 – Always	0.56

Additional file 2: Continued

Index name and (No. of items) and Component items	Range of possible scores	Cronbach's α
**Sun safety and/or skin cancer prevention programs** (3 items)<br><br>In your aquatic programs, indicate whether you provide each of these types of programs about sun safety and/or skin cancer prevention:<br>1. Programs/policies for lifeguards<br>2. Programs/policies for swimmers<br>3. Educational activities in swimming lessons	Mean of nonmissing items when at least half of the items were answered<br><br>Answers:<br>1 – Rarely or never<br>2 – Sometimes<br>3 – Usually<br>4 – Always	0.75
**Sun safety environments and policies** (4 items)<br><br>This summer, did your pool…<br>1. Encourage swimmers to stay in the shade when they're not swimming?<br>2. Remind children to wear sunscreen?<br>3. Remind parents to send children with sunscreen?<br>4. Provide sunscreen for swimmers who forgot to put it on beforehand?	1-4<br><br>Answer: Yes/No<br>Index score: sum of yes answers<br><br>Recode of index score:<br>0,1 = 1<br>2 = 2<br>3 = 3<br>4 = 4	0.73
