# Supplementary material for: Individual and setting level predictors of the implementation of a skin cancer prevention program: a multilevel analysis
Source: Implement Sci. 2010 May 31;5:40. doi: 10.1186/1748-5908-5-40 (PMC2901365; doi:10.1186/1748-5908-5-40)
Supplement: Additional file 3 — Multilevel model results with Level 1 predictors for 2004. This pdf file provides the coefficient estimates and other model-related information for the sub-models (Models 1 through 4) created using level 1 domains. [file 1748-5908-5-40-S3.PDF]

Additional file 3: Lifeguard-level predictors based on four models of Lifeguard Pool Cool participation for 2004 analysis

Variable	Model 1		Model 2		Model 3		Model 4	
	**Coefficient**	**p value**	**Coefficient**	**p value**	**Coefficient**	**p value**	**Coefficient**	**p value**
**Intercept**	4.267	0.000	4.297	0.000	4.309	0.000	4.151	0.000
**Lifeguard-level predictors**								
**Lifeguard background characteristics**								
Female	0.226	0.011	0.203	0.023	0.214	0.016	0.208	0.017
Age	0.021	0.083	0.019	0.116	0.021	0.072	0.021	0.073
At least some college education	0.549	0.000	0.535	0.000	0.463	0.000	0.464	0.000
Caucasian	-0.106	0.473	-0.119	0.420	-0.135	0.359		
Skin cancer risk	-0.031	0.584	-0.031	0.586	-0.021	0.710		
**Lifeguard sun protection-related attitudes**								
Sun protective benefits			0.280	0.002	0.220	0.012	0.215	0.014
Sun protective barriers			0.002	0.982	0.020	0.768	0.019	0.787
Sun protective norms			0.113	0.062	0.087	0.167	0.090	0.149
**Lifeguard sun protection-related behaviors**								
Sun protective behaviors					0.231	0.006	0.228	0.006
Sun exposure					0.143	0.000	0.144	0.000
	**Deviance**	**Param**	**Deviance**	**Param**	**Deviance**	**Param**	**Deviance**	**Param**
Model fit	11737.13	8	11718.86	11	11687.67	13	11689.10	11
	**AIC**		**AIC**		**AIC**		**AIC**	
	11753.13		11740.86		11713.67		11711.1	
